# The longitudinal relationship between immune cell profiles and frailty in patients with breast cancer receiving chemotherapy

**DOI:** 10.1186/s13058-021-01388-w

**Published:** 2021-02-05

**Authors:** Nikesha Gilmore, Supriya Mohile, Lianlian Lei, Eva Culakova, Mostafa Mohamed, Allison Magnuson, Kah Poh Loh, Ronald Maggiore, Elizabeth Belcher, Alison Conlin, Lora Weiselberg, Mary Ontko, Michelle Janelsins

**Affiliations:** 1 Cancer Control, University of Rochester School of Medicine and Dentistry, Rochester, New York USA; 2 James P. Wilmot Cancer Institute, University of Rochester School of Medicine and Dentistry, Rochester, New York USA; 3 Department of Psychiatry, University of Michigan, Ann Arbor, Michigan USA; 4 Pacific Cancer Research Consortium NCORP, Providence Cancer Institute Franz Clinic, Portland, Oregon USA; 5 Northwell Health NCORP, The Monter Cancer Center, Lake Success, New York, USA; 6 Dayton Clinical Oncology Program, Dayton, Ohio USA

**Keywords:** Frailty, Chemotherapy, Inflammation, Breast cancer, Immune cell profiles, Cellular markers of inflammation

## Abstract

**Background:**

Frailty is associated with an increased risk of chemotherapy toxicity. Cellular markers of inflammation can help identify patients with frailty characteristics. However, the role of cellular markers of inflammation in identifying patients at risk of developing chemotherapy-induced frailty and their clinical utility are not fully understood.

**Methods:**

This study was a secondary analysis of a large nationwide cohort study of women with stage I–IIIC breast cancer (*n* = 581, mean age 53.4; range 22–81). Measures were completed pre-chemotherapy (T1), post-chemotherapy (T2), and 6 months post-chemotherapy (T3). Frailty was assessed at all three time points using a modified Fried score consisting of four self-reported measures (weakness, exhaustion, physical activity, and walking speed; 0–4, 1 point for each). Immune cell counts as well as neutrophil to lymphocyte ratio (NLR) and lymphocyte to monocyte ratio (LMR) were obtained at T1 and T2 time points. Separate linear regressions were used to evaluate the associations of (1) cell counts at T1 with frailty at T1, T2, and T3 and (2) change in cell counts (T2–T1) with frailty at T2 and T3. We controlled for relevant covariates and frailty at the T1 time point.

**Results:**

From T1 to T2, the mean frailty score increased (1.3 vs 2.0; *p* < 0.01) and returned to T1 levels by the T3 time point (1.3 vs 1.3; *p* = 0.85). At the T1 time point, there was a positive association between cellular markers of inflammation and frailty: WBC (*β* = 0.04; *p* < 0.05), neutrophils (*β* = 0.04; *p* < 0.05), and NLR (*β* = 0.04; *p* < 0.01). From T1 to T2, a greater increase in cellular markers of inflammation was associated with frailty at T2 (WBC: *β* = 0.02, *p* < 0.05; neutrophils: *β* = 0.03, *p* < 0.05; NLR: *β* = 0.03; *p* < 0.01). These associations remained significant after controlling for the receipt of growth factors with chemotherapy and the time between when laboratory data was provided and the start or end of chemotherapy.

**Conclusions:**

In patients with breast cancer undergoing chemotherapy, cellular markers of inflammation are associated with frailty. Immune cell counts may help clinicians identify patients at risk of frailty during chemotherapy.

**Trial registration:**

ClinicalTrials.gov, NCT01382082

**Supplementary Information:**

The online version contains supplementary material available at 10.1186/s13058-021-01388-w.

## Background

Breast cancer is one of the most commonly diagnosed cancers in women in the USA [1]. Fortunately, mortality rates due to breast cancer have been on a steady decline over the last decade [1]. Despite evidence that adjuvant chemotherapy for breast cancer reduces the risk of disease recurrence, these treatments have concomitant consequences and may cause side effects that are associated with frailty [2]. Frailty categorizes an individual’s physiologic reserves and is a significant problem for patients with breast cancer as well as survivors of cancer and particularly for long-term survivors of pediatric malignancies [3]. Fried and colleagues defined frailty phenotypically as a clinical syndrome consisting of three or more of the following: weakness, fatigue, low physical activity, slow walking speed, and unintentional weight loss [4]. The association of frailty with a variety of adverse outcomes has been well established. Frailty is associated with an increased vulnerability to stressors, impaired cognitive function, and increased risk of disability and mortality [4–7]. Furthermore, it has been demonstrated that frail women with breast cancer have an increased risk of chemotherapy toxicity, reduced chemotherapy tolerance, and lower health-related quality of life [8, 9]. Given the adverse effects of frailty on outcomes in patients with cancer, understanding the factors that contribute to frailty as well as determining which biological markers can identify the women with breast cancer who are at increased risk of chemotherapy-induced frailty and may improve clinical outcomes.

Chronic inflammation has been shown to contribute to the development of frailty [10]. Leukocytes (white blood cells; WBC), an essential part of the immune system, consist of granulocytes (neutrophils, basophils, and eosinophils), lymphocytes (T, B, and NK cells), and monocytes (macrophages and dendritic cells). An increase in the relative numbers of different types of leukocytes is an indicator of systemic inflammation. Although there are more precise ways to measure inflammation, such as the use of biochemical markers, these measures are time consuming, expensive, and not typically part of routine clinical practice. Thus, total and differential counts of cellular markers of inflammation such as neutrophils, lymphocytes, monocytes, neutrophil to lymphocyte ratio (NLR), and lymphocyte to monocyte to ratio (LMR) are commonly used because these are frequently measured as part of clinical care [11, 12]. These cellular markers of inflammation are elevated in frail community-dwelling older women [13]. Specifically, increased counts of circulating total WBC, neutrophils, and monocytes have been shown to be associated with frailty [13, 14]. These associations have also been demonstrated in patients with cancer; a high NLR is positively associated with frailty and is also associated with reduced physical and functional outcomes prior to starting cancer treatment [15]. Cellular markers of inflammation have also been reported to have robust prognostic value in a variety of cancers. For instance, increased total WBC, neutrophils, and monocytes and decreased lymphocytes are associated with increased mortality [13, 16–21], and elevated NLR and lower LMR are predictive of poor prognosis [12, 22, 23].

Taken together, these studies have improved our understanding of the biochemical and cellular markers of inflammation that are associated with frailty in community-dwelling adults as well as in patients with cancer. However, the longitudinal relationship between total and differential leukocytes, in particular neutrophils, lymphocytes, monocytes, NLR, and LMR, prior to receiving chemotherapy with acute and persistent frailty after chemotherapy, has not yet been described. To address these questions in patients with breast cancer receiving chemotherapy, we investigated whether pre-chemotherapy levels of these cellular markers of inflammation as well as their change with chemotherapy were associated with post-chemotherapy frailty and frailty that persists up to 6 months after the completion of chemotherapy. We hypothesized that patients with a heightened inflammatory state, as evidenced by an imbalance in their cellular markers of inflammation prior to receiving chemotherapy, and those with the greatest increase in their cellular markers of inflammation with chemotherapy would be more likely to develop chemotherapy-induced frailty.

## Methods

### Study design

**Fig. 1:**
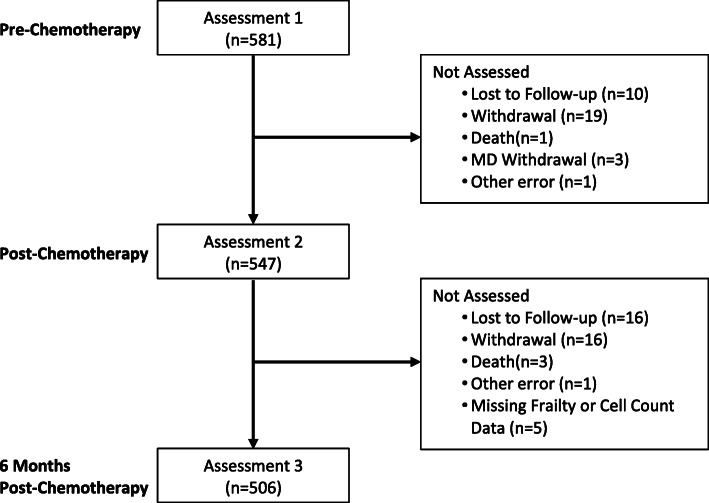
Consort diagram

We conducted a secondary analysis of data from a large nationwide prospective, cohort study that has been previously published [24, 25]. We investigated the association of cellular markers of inflammation with frailty in female patients with breast cancer. Patients were recruited throughout the USA from the University of Rochester Cancer Center (URCC) National Cancer Institute (NCI) Community Oncology Research Program (NCORP) Community Affiliates. The primary study was a longitudinal cohort study aimed to determine the effects of chemotherapy on cognition (URCC 10055; ClinicalTrials.gov identifier NCT01382082) and enrolled participants with breast cancer and paired controls from 2011 to 2013 [24, 25]. Measures were completed within 7 days of the first cycle of chemotherapy (pre-chemotherapy), within 4 weeks of the last chemotherapy cycle (post-chemotherapy), and 6 months after the last chemotherapy cycle. For this study, we included all patients with breast cancer with available total and differential leukocyte data (Fig. 1). Institutional review boards at the URCC NCORP Research Base and at each of the NCORP Community Affiliates approved the study. All participants provided informed consent before completing study requirements.

### Study participants

In the primary study, patients were included if they were (1) female, (2) diagnosed with invasive breast cancer (stage I–IIIC) and scheduled to begin a course of adjuvant or neoadjuvant chemotherapy, (3) chemotherapy naïve, (4) expected to live greater than 10 months, (5) able to speak and read English, and (6) able to provide written informed consent. Patients were excluded if they were (1) hospitalized at the time of study or had been hospitalized within the last year for a psychiatric illness, (2) diagnosed with a neurodegenerative disease, (3) had any primary central nervous system disease, (4) scheduled to receive concurrent radiation treatment while receiving chemotherapy, (5) have (or have had) metastatic disease and pregnant, and/or (6) colorblind [24].

### Measures

#### Frailty

Our primary outcome measures were frailty determined pre-chemotherapy (within 7 days of the first cycle of chemotherapy), post-chemotherapy (within 4 weeks of the last chemotherapy cycle), and 6-month post-chemotherapy time points. Fried’s frailty score was used to assess frailty in this cohort. Fried’s frailty is a validated measure used to identify frail individuals by assessing five criteria: weakness, exhaustion, walking speed, physical activity, and unintentional weight loss. The primary study (URCC10055) did not include measures to assess unintentional weight loss. As a result, we used a modified version of Fried’s frailty score using four available criteria (weakness, exhaustion, walking speed, and physical activity) as has been previously reported [26, 27]. The four criteria were assessed using self-reported validated measures as follows: (1) weakness (≥ 4 on scale of 1–10 on Symptom Inventory (SI)); (2) exhaustion (≥ 4 on scale of 1–10 on SI); (3) walking speed (< 2 mph on Aerobic Center Longitudinal Study Physical Activity Questionnaire (ACLS)), and physical activity (< 150 min/week on ACLS) [26–28]. For each criterion, participants received a score of 1 if they met the cut-off for that criterion; otherwise, they received a score of 0. Thus, participants received a frailty score ranging from 0 to 4, with a score of 0 having the least frailty characteristics and four having the most frailty characteristics.

#### Immune cell composition

Laboratory data taken from the medical record were also obtained at the pre-chemotherapy and post-chemotherapy time points. Pre-chemotherapy laboratory data were obtained on average 12.2 (SD = 17.8) days before the first day of chemotherapy (with approximately 70% of laboratory data obtained within 14 days of the start of chemotherapy). Post-chemotherapy laboratory data were obtained on average 18.4 (SD = 24.3) days after the last day of chemotherapy (with approximately 70% of laboratory data obtained within 21 days of the end of chemotherapy). Total WBC, neutrophils, lymphocytes, monocytes, and platelets were reported in 10^3^ cells/μL and extracted from the medical records by study staff. Hemoglobin and albumin reported in grams per deciliter and hematocrit reported in percentages were also extracted from the medical records. A physician with board certifications in hematology and geriatric medicine reviewed all lab values to confirm reporting accuracy. Any discrepancies were clarified by community-affiliated sites according to standard operating procedures. NLR and LMR were calculated. Neutrophils, lymphocytes, monocytes, NLR, LMR, and total WBC were assessed as our independent variables.

### Statistical analyses

Participants self-reported age, race, education, and marital status. Age was categorized into < 50, 50–64, and ≥ 65 years. Other demographic variables were dichotomized accordingly: race (white vs non-white), education (high school or below vs some college or above), and marital status (married/long-term relationship vs others). Cancer stage, treatment type (adjuvant vs neoadjuvant), type of chemotherapy (anthracycline vs non-anthracycline), growth factor treatment (yes vs no), date that the complete blood count (CBC) was obtained, and the start and end dates of chemotherapy were extracted from the medical records. Descriptive statistics were used to summarize the baseline characteristics of the sample.

Chi-square tests were used to compare differences in the components of frailty and frailty score between pre-chemotherapy and post-chemotherapy and between pre-chemotherapy and 6 months post-chemotherapy. Paired *T* tests were used to compare the differences in neutrophils, lymphocytes, monocytes, NLR, LMR, total WBC, hematocrit, hemoglobin, albumin, and platelets between pre-chemotherapy and post-chemotherapy and between pre-chemotherapy and 6 months post-chemotherapy. Separate linear regression models were used to evaluate the relationships between (1) pre-chemotherapy cellular markers of inflammation (neutrophils, lymphocytes, monocytes, NLR, LMR, and total WBC) and pre-chemotherapy frailty; (2) pre-chemotherapy cellular markers of inflammation and post-chemotherapy frailty; (3) pre-chemotherapy cellular markers of inflammation and 6-month post-chemotherapy frailty; (4) change in cellular markers of inflammation (from pre-chemotherapy to post-chemotherapy) and post-chemotherapy frailty; and (5) change in cellular markers of inflammation (from pre-chemotherapy to post-chemotherapy) and 6 months post-chemotherapy. In each model, we controlled for baseline frailty, age, race, marital status, and education.

Given that chemotherapy reduces individuals’ total WBC, hematopoietic growth factors are commonly given to patients with breast cancer undergoing chemotherapy as supportive care medications to assist in the production of new WBC to prevent infection. Due to the effect of growth factors (given 24–48 h after chemotherapy infusion) on the WBC counts, we wanted to rule out the influence of growth factors on the association of cellular markers of inflammation with frailty after the completion of chemotherapy. Thus, all multivariate analyses were repeated in the subset of patients who received growth factors support after chemotherapy infusions. We did not evaluate the influence of the timing of steroids on the association of cellular markers of inflammation with frailty at the pre-chemotherapy time point as steroids are routinely given within 30 min of the chemotherapy infusion (i.e., after laboratories are obtained).

Furthermore, given that the timing of the date that the laboratory data was variable within the allotted study time point windows and the start and end dates of chemotherapy could affect immune cell counts as well as their ratios, we wanted to determine the influence (if any) of the timing of the cell counts and the start and end date of chemotherapy on the association of immune cell profiles and frailty. Thus, all multivariate analyses were repeated controlling for either the number of days between the pre-chemotherapy blood draw and the start of chemotherapy or the number of days between the post-chemotherapy blood draw and the last day of chemotherapy as appropriate.

Analyses were performed using either SAS v. 9.4 (SAS Institute, Cary, NC) or JMP Pro v. 15 (SAS Institute, Cary, NC). *p* < 0.05 was used to assess statistical significance.

## Results

### Patient and clinical characteristics

A summary of participants’ baseline demographic and clinical characteristics is shown in Table 1. All female patients with breast cancer from the primary study were included in the analysis [24, 25]. A total of 581 patients was included in this analysis (Fig. 1). The mean age of the patients was 53.4 (range 22–81; SD 10.6 years). The majority of patients were white (89.2%), received some college education (75.6%), and were married or in a long-term relationship (72.6%). Eighty-three percent of patients received adjuvant treatment for their cancer and 27.2% had stage I, 49.2% had stage II, and 18.6% had stage III breast cancer. Most patients received growth factors with at least one cycle of treatment (81.1%) and 47.9% were treated with an anthracycline.

**Table 1:** Baseline demographic and clinical characteristics

		Frailty score				
		0	1	2	3	4
Total participants: *N* = 581	***N =*** 581	***N*** = 173	***N*** = 195	***N*** = 109	***N*** = 88	***N*** = 16
**Age, years:** ***N*** **(%)**						
Mean [range]	53.4 [22–81]	54.3 [29–72]	54.3 [22–76]	51.7 [26–75]	51.3 [26–81]	55.75 [35–81]
< 50	205 (35.3)	57 (32.9)	58 (29.7)	46 (42.2)	39 (44.3)	5 (31.3)
50–64	284 (48.9)	87 (50.3)	101 (51.8)	49 (45.0)	39 (44.3)	8 (50.0)
≥ 65	92 (15.8)	29 (16.8)	36 (18.5)	14 (12.8)	10 (11.4)	3 (18.7)
**Race:** ***N*** **(%)**						
White	518 (89.2)	158 (91.3)	178 (91.3)	94 (86.2)	73 (83.0)	15 (93.8)
Non-white	63 (10.8)	15 (8.7)	17 (8.7)	15 (13.8)	15 (17.0)	1 (6.2)
**Education:** ***N*** **(%)**						
High school or below	142 (24.4)	37 (21.4)	42 (21.5)	30 (27.5)	27 (30.7)	6 (37.5)
Some college or above	439 (75.6)	136 (78.6)	153 (78.5)	79 (72.5)	61 (69.3)	10 (62.5)
**Marital status:** ***N*** **(%)**						
Married/long-term relationship	422 (72.6)	139 (80.3)	139 (71.3)	71 (65.1)	66 (75.0)	7 (43.8)
Others	159 (27.4)	34 (19.7)	56 (28.7)	38 (34.9)	22 (25.0)	9 (56.2)
**Cancer stage:** ***N*** **(%)**						
I	158 (27.2)	50 (28.9)	59 (30.3)	27 (24.8)	18 (20.4)	4 (25.0)
II	286 (49.2)	94 (54.3)	85 (43.6)	56 (51.4)	44 (50.0)	7 (43.8)
III	108 (18.6)	24 (13.9)	42 (21.5)	20 (18.3)	18 (20.5)	4 (25.0)
Unknown	29 (5.0)	5 (2.9)	9 (4.6)	6 (5.5)	8 (9.1)	1 (6.2)
**Type of treatment:** ***N*** **(%)**						
Adjuvant	481 (82.8)	145 (83.8)	154 (79.0)	92 (84.4)	76 (86.4)	14 (87.5)
Neoadjuvant	100 (17.2)	28 (16.2)	41 (21.0)	17 (15.6)	12 (13.6)	2 (12.5)
**Type of chemotherapy:** ***N*** **(%)**						
Anthracycline	278 (47.9)	85 (49.1)	91 (46.7)	57 (52.2)	38 (43.2)	7 (43.8)
Non-anthracycline	303 (52.1)	88 (50.9)	104 (53.3)	52 (47.8)	50 (56.8)	9 (56.2)
**Growth factor:** ***N*** **(%)**						
Yes	471 (81.1)	147 (85.0)	152 (77.9)	89 (81.7)	70 (79.5)	13 (81.3)
No	110 (18.9)	26 (15.0)	43 (22.1)	20 (18.3)	18 (20.5)	3 (18.7)

The mean frailty score of patients before receiving chemotherapy was 1.3 (SD 1.1; Table 2). The frailty score increased significantly to 2.0 (SD 1.2) post-chemotherapy and returned to pre-chemotherapy levels (mean score = 1.3; SD 1.1) by 6 months post-chemotherapy (Table 2). In post-chemotherapy compared to pre-chemotherapy, patients reported that they were weaker (54.3% vs 25.0%; *p* < 0.001), more exhausted (63.9% vs 39.7%; *p* = 0.008), walked more slowly (66.6% vs 54.5%; *p* < 0.001), and engaged in less physical activity (14.3% vs 9.2%; *p* = 0.008) (Table 2). Six months after the completion of chemotherapy, patients were less active (completed < 150 min/week of physical activity) than they were pre-chemotherapy (6.0% vs 9.2%; *p* = 0.049) (Table 2). All other components of frailty returned to pre-chemotherapy levels 6 months after the completion of their chemotherapy regimen (Table 2).

**Table 2:** Difference in frailty and immune cell profiles pre-, post-, and 6 months post-chemotherapy

	Pre-chemo *N* (%)	Post-chemo *N* (%)	6 months post-chemo *N* (%)	Pre- vs post-chemo (*p* value)	Pre- vs 6 months post-chemo (*p* value)
**Frailty components**					
Weakness ≥ 4	145 (25.0)	290 (54.3)	153 (30.2)	**< 0.001**	0.056
Exhaustion ≥ 4	230 (39.7)	342 (63.9)	214 (42.3)	**0.008**	0.378
Walk speed < 2 mph	313 (54.5)	353 (66.6)	254 (50.7)	**< 0.001**	0.210
Physical activity < 150 min/week	53 (9.2)	76 (14.3)	30 (6.0)	**0.008**	**0.049**
**Frailty score:** *N* (%)					
0	173 (29.8)	74 (13.8)	154 (30.4)	**< 0.001**	0.480
1	195 (33.6)	122 (22.8)	151 (29.8)		
2	109 (18.8)	124 (23.1)	115 (22.7)		
3	88 (15.2)	173 (32.3)	74 (14.6)		
4	16 (2.8)	43 (8.0)	12 (2.37)		
Mean [SD]	1.3 [1.1]	2.0 [1.2]	1.3 [1.1]	**< 0.001**	0.846
**Cell counts:** mean [SD]					
Neutrophils (10^3^ cells/μL)	4.69 [2.68]	4.84 [5.01]	–	0.560	–
Lymphocytes (10^3^ cells/μL)	1.88 [0.97]	1.22 [1.48]	–	**< 0.0001**	–
Monocytes (10^3^ cells/μL)	0.53 [0.55]	0.54 [0.53]	–	0.947	–
NLR	3.00 [2.98]	5.03 [5.71]	–	**< 0.001**	–
LMR	5.72 [31.2]	2.8 [1.98]	–	**0.038**	–
Total WBC (10^3^ cells/μL)	7.42 [3.05]	6.59 [4.76]	–	**< 0.001**	–
Hemoglobin (g/dL)	12.67 [2.28]	11.12 [1.58]	–	**< 0.001**	–
Hematocrit (%)	37.50 [6.92]	33.26 [4.65]	–	**< 0.001**	–
Platelets (10^3^ cells/μL)	260.27 [81.82]	237.93 [89.16]	–	**< 0.001**	–
Albumin (g/dL)	4.17 [0.43]	3.93 [0.47]	–	**< 0.001**	–

*SD* standard deviation, *μL* microliter, *g* gram, *dL* deciliter, *%* percent, *NLR* neutrophil to lymphocyte ratio, *LMR* lymphocyte to monocyte ratio, *WBC* white blood cell

### Cellular inflammatory markers, frailty, and chemotherapy

In pre-chemotherapy compared to post-chemotherapy, there was a significant increase in NLR (3.00 vs 5.03; *p* < 0.001), with a significant decrease in the mean cell count of lymphocytes (1.88 vs 1.22; *p* < 0.001), total WBC count (7.42 vs 6.59; *p* < 0.001), and LMR (5.72 vs 2.80; *p* = 0.038) (Table 2). There was no significant change in levels of neutrophils or monocytes with chemotherapy (Table 2).

In multivariate analyses, total WBC (*β* = 0.037; *p* < 0.05), neutrophils (*β* = 0.041; *p* < 0.5), and NLR (*β* = 0.044; *p* < 0.01) at the pre-chemotherapy time point were associated with pre-chemotherapy frailty (Table 3). We repeated these analyses controlling for the number of days between the pre-chemotherapy blood draw and the start of chemotherapy; we found that these associations remained significant (total WBC (*β* = 0.039; *p* < 0.05), neutrophils (*β* = 0.39; *p* < 0.05), and NLR (*β* = 0.041; *p* < 0.05); Supplementary Table 1) regardless of the timing of the collection of pre-chemotherapy cell counts.

**Table 3:** Association of pre-chemotherapy cell counts and pre-chemotherapy frailty

Pre-chemotherapy	Pre-chemotherapy frailty score *β* (SE)					
Neutrophils	0.041**					
	**(0.018)**					
Lymphocytes		0.043				
		(0.051)				
Monocytes			0.069			
			(0.090)			
NLR				0.044***		
				**(0.017)**		
LMR					0.001	
					(0.002)	
WBC						0.037**
						**(0.015)**
Age 50–64	− 0.134	− 0.132	− 0.147	− 0.132	− 0.134	− 0.151
	(0.109)	(0.109)	(0.109)	(0.111)	(0.111)	(0.104)
Age 65+	− 0.298*	− 0.289*	− 0.281*	− 0.276*	− 0.275*	− 0.270*
	(0.153)	(0.154)	(0.153)	(0.156)	(0.155)	(0.146)
White (yes = 1)	− 0.272*	− 0.261	− 0.230	− 0.313*	− 0.273	− 0.256*
	(0.162)	(0.162)	(0.160)	(0.165)	(0.167)	(0.153)
Married (yes = 1)	− 0.261**	− 0.245**	− 0.250**	− 0.283**	− 0.280**	− 0.248**
	(0.113)	(0.114)	(0.113)	(0.115)	(0.115)	(0.109)
Some college or above = 1	− 0.271**	− 0.226*	− 0.233**	− 0.259**	− 0.220*	− 0.268**
	(0.116)	(0.116)	(0.116)	(0.118)	(0.118)	(0.110)
Constant	1.846***	1.902***	1.929***	1.955***	2.006***	1.730***
	(0.210)	(0.228)	(0.201)	(0.203)	(0.200)	(0.221)
Observations	513	516	517	499	495	553
*R*-squared	0.043	0.032	0.031	0.047	0.033	0.044

Linear regression models were used to evaluate the association between cell counts and frailty, controlling for age (below 50, 50–64 versus ≥ 65 years), race (Caucasian versus others), marital status (married versus others), and education (≥ some college versus ≤ high school)

*NLR* neutrophil to lymphocyte ratio, *LMR* lymphocyte to nonocyte ratio

*Significant at 10%; **significant at 5%; ***significant at 1%

Multivariate analyses also demonstrated that a greater increase (from pre-chemotherapy to post-chemotherapy) in total WBC (*β* = 0.024; *p* < 0.05), neutrophils (*β* = 0.026; *p* < 0.05), and NLR (*β* = 0.032; *p* < 0.01) was associated with frailty at the post-chemotherapy time point (Table 4). Similarly, we repeated these analyses controlling for the number of days between the post-chemotherapy blood draw and the last day of chemotherapy; we also found that these associations remained significant (total WBC (*β* = 0.021; *p* < 0.05), neutrophils (*β* = 0.24; *p* < 0.05), and NLR (*β* = 0.029; *p* < 0.01); Supplementary Table 2) regardless of the timing of the collection of post-chemotherapy cell counts.

**Table 4:** Association of change in cell counts (pre- to post-chemotherapy) and with post-chemotherapy frailty

Change in cell counts	Post-chemotherapy frailty score: *β* (SE)					
Neutrophils	0.026**					
	**(0.010)**					
Lymphocytes		0.042				
		(0.032)				
Monocytes			0.019			
			(0.074)			
NLR				0.032***		
				**(0.009)**		
LMR					− 0.003*	
					(0.002)	
WBC						0.024**
						**(0.009)**
Baseline frailty	0.318***	0.312***	0.308***	0.332***	0.321***	0.297***
	(0.046)	(0.047)	(0.047)	(0.047)	(0.048)	(0.045)
Age 50–64	0.216*	0.224*	0.215*	0.210*	0.211*	0.212*
	(0.115)	(0.115)	(0.115)	(0.116)	(0.119)	(0.111)
Age 65+	0.312*	0.323**	0.301*	0.270	0.332**	0.240
	(0.160)	(0.161)	(0.161)	(0.164)	(0.167)	(0.156)
White (yes = 1)	0.104	0.145	0.083	0.091	0.143	0.036
	(0.176)	(0.177)	(0.174)	(0.179)	(0.183)	(0.169)
Married (yes = 1)	− 0.331***	− 0.322***	− 0.328***	− 0.281**	− 0.268**	− 0.311***
	(0.118)	(0.120)	(0.120)	(0.121)	(0.124)	(0.116)
Some college or above = 1	0.152	0.136	0.113	0.204	0.143	0.185
	(0.123)	(0.123)	(0.123)	(0.125)	(0.127)	(0.118)
Constant	1.444***	1.462***	1.520***	1.296***	1.350***	1.564***
	(0.230)	(0.233)	(0.228)	(0.236)	(0.239)	(0.223)
Observations	459	459	461	439	427	508
*R*-squared	0.135	0.123	0.115	0.147	0.126	0.115

Linear regression models were used to evaluate the association between cell counts and frailty, controlling for age (below 50, 50–64 versus ≥ 65 years), race (white versus non-white), marital status (married/long term relationship versus others), and education (≥ some college versus ≤ high school)

*NLR* neutrophil to lymphocyte ratio, *LMR* lymphocyte to monocyte ratio

*Significant at 10%; **significant at 5%; ***significant at 1%

We next tested the association of cellular markers of inflammation with frailty in the subset of participants who received growth factors with chemotherapy and found that these associations remained significant (total WBC (*β* = 0.023; *p* < 0.05), neutrophils (*β* = 0.023; *p* < 0.05), and NLR (*β* = 0.031; *p* < 0.01); Supplementary Table 3).

In these models, higher pre-chemotherapy frailty and being not being married or in a long-term relationship were predictive of post-chemotherapy frailty. No significant associations were found between pre-chemotherapy cell counts and post-chemotherapy frailty score (Supplementary Table 4) or in the subset of patients who received growth factors with chemotherapy (Supplementary Table 5). There were also no significant associations between change in cell count from pre-chemotherapy to post-chemotherapy and 6-month post-chemotherapy frailty score (Supplementary Table 6).

## Discussion

In this secondary analysis of data from a nationwide, multi-center longitudinal cohort study, we confirmed the relationships between inflammation and frailty characteristics that have been reported in community-dwelling older adults as well as older adults with cancer over the age of 65 years [12, 14, 21]. Additionally, we demonstrated an association between cellular markers of inflammation and chemotherapy-induced frailty characteristics. We showed that a greater increase in neutrophils, NLR, and total WBC from pre-chemotherapy to post-chemotherapy was positively associated with frailty characteristics at the post-chemotherapy time point. Moreover, we demonstrated that these associations were not affected by the receipt of growth factors with chemotherapy or by the time between when the laboratory data was obtained and the start or end of chemotherapy. In this cohort of patients, we found that in women with breast cancer undergoing chemotherapy, frailty increased from pre-chemotherapy to post-chemotherapy and returned to pre-chemotherapy levels by 6 months after the completion of chemotherapy. To our knowledge, this is the first study to investigate the predictive effect of cellular markers of inflammation using laboratory data on frailty characteristics that are associated with chemotherapy.

Chronic low-grade inflammation (i.e., subclinical inflammation) is independently associated with frailty. Leukocytes are an essential component of the immune system and consist of neutrophils, eosinophils, and basophils (that comprise the granulocytic component) as well as lymphocytes and monocytes (that comprise the non-granulocytic component). Neutrophils are the most abundant granulocytic leukocyte and have traditionally been thought of as the first line of defense against infections. However, recent studies have shown that neutrophils also play a vital role in chronic inflammatory responses in immunological diseases such as cancer by interacting with other immune cells such as lymphocytes [29]. We have shown that in patients with breast cancer, the level of neutrophils in peripheral blood is independently associated with frailty characteristics prior to receiving chemotherapy.

While no significant changes were observed in the levels of neutrophils and monocytes from pre-chemotherapy to post-chemotherapy, there were significant changes in the concentration of WBC, lymphocytes, NLR, LMR, hemoglobin, hematocrit, platelets, and albumin. However, it is important to emphasize that these changes remained within the laboratory-referenced normal range. Given that these tests were completed within 1 month of completing chemotherapy and that the values remained within the normal ranges suggests that the hematopoietic systems of these patients recovered after chemotherapy.

Notably, even though there were subtle and non-significant changes within the normal range of neutrophil concentration from pre-chemotherapy to post-chemotherapy, we found that an increase in neutrophils from pre-chemotherapy to post-chemotherapy was associated with frailty at the post-chemotherapy time point, after controlling for the pre-chemotherapy frailty score. These findings suggest that subclinical changes (changes within the normal range) are important to consider when making treatment decisions based on the effect of cancer treatment on frailty in patients with breast cancer.

Along with neutrophilic responses, NLR is also commonly used as a marker of subclinical inflammation. NLR can signify an imbalance between various components of the immune system, with higher neutrophils indicating an activation of the pro-inflammatory immune pathways and lower lymphocytes reflecting depressed cellular immunity [11]. In fact, in patients with breast cancer, an elevated NLR has been associated with poor prognosis [30, 31]. The observed independent association of NLR with frailty suggests that low-grade inflammation as well as subclinical changes in inflammation is a prognostic factor for frailty in patients with breast cancer. Unfortunately, the mechanism underlying the contribution of NLR to poor outcomes including frailty is unknown. Emerging evidence indicates that the roles of neutrophils are more complex than previously thought. In patients with cancer, neutrophils can be polarized to exhibit different phenotypes depending on which tumor-derived factors as well as other immune cells interact with them. As a result, in patients with cancer, neutrophils can have either immunostimulating or immunosuppressive properties. Immunostimulating neutrophils, also known as anti-tumorigenic neutrophils, can activate cytotoxic CD8+ T cells (a subset of lymphocytes) to exert immunostimulating effects [29, 32]. On the other hand, in patients with cancer, neutrophils have been shown to exert immunosuppressive properties [33, 34]. More work is needed to clarify the varying roles of neutrophil subsets as well as the ratios of different leukocyte subsets as immunological biomarkers that may predict frailty and frailty trajectories in patients with breast cancer.

A recent study by Bailur et al. aimed to elucidate the association of distinct immune subsets with frailty through the use of immunoprofiling flow cytometry techniques. These authors showed that in older adults with breast cancer, patients with higher pre-chemotherapy levels of granulocytic cells but lower levels of myeloid-suppressor cells and regulatory T cells were more frail prior to initiating chemotherapy [35]. While no association was found between pre-chemotherapy immune subsets and frailty (assessed using the geriatric assessment) at 3 and 12 months after starting chemotherapy, these profiles were predictive of unexpected hospitalizations. Our study had complementary findings; we demonstrated that neutrophils, which make up a major component of the granulocytic immune component, were associated with frailty in patients with cancer. While Bailur et al. found no association between immune cell profiles and frailty after chemotherapy, we found that changes to the neutrophil component following chemotherapy were independently associated with post-chemotherapy frailty but not with frailty 6 months after completing chemotherapy. These differences in findings may be multifactorial. Firstly, participants in our study were younger (mean age 53 vs 73). Secondly, there were differences in the timing of blood draws for the post-chemotherapy time point (within 4 weeks after the last chemotherapy cycle (mean 18.4 (SD = 24.3) days) vs on the day of the last cycle of chemotherapy). Thirdly, in the current study, we examined the association between longitudinal changes in cellular markers of inflammation over the course of treatment and frailty. Nevertheless, the study by Bailur et al. indicates that chemotherapy has varying effects on different cell subsets within the immune system. Thus, when assessing the role of immune cell components on clinical outcomes of patients with cancer in the context of chemotherapy, the change of each immune subset should be carefully monitored. Together our current study along with that of Bailur et al. sheds light on the complexity of immunological biomarkers as predictors of frailty. Future studies evaluating the value of immune cell subsets as biomarkers to predict frailty should consider immunoprofiling techniques, where the individual contributions of distinct immune subsets (e.g., immunostimulating vs immunosuppressive neutrophils) are evaluated for their association with chemotherapy-induced frailty.

Interestingly, while the changes in neutrophils and NLR were associated with post-chemotherapy frailty as well as change in frailty from pre-chemotherapy to post-chemotherapy, they were not predictive of frailty 6 months after completing chemotherapy. This suggests that while immune cell counts might be valuable in providing information to assist oncologists in making decisions about acute frailty, these immune profiles might not be predictive of chronic chemotherapy-induced frailty in patients with breast cancer.

Although frailty is typically characterized as an aging-related condition, it is important to recognize that frailty also exists in younger patients, especially in the context of cancer [3, 36]. It has been demonstrated that childhood survivors of cancer are more frail than their aged-matched non-cancer counterparts, and they exhibit features of accelerated aging [3]. Cancer contributes to biological changes across varying domains that result in an overall dysregulation of energy systems. This dysregulation has clinical manifestations such as weakness, exhaustion, low physical activity, slow walking speed, and weight loss which ultimately constitutes the frailty phenotype [4, 37]. Thus, in patients with cancer, frailty is a stronger predictor than age of negative cancer treatment outcomes such as post-operative outcomes, chemotherapy-related toxicities, unexpected hospitalizations, morbidity, and mortality [2]. In our cohort, we found that patients with breast cancer with a mean age of 53 years were already exhibiting frailty characteristics prior to starting chemotherapy, with about 40% of patients having more than two frailty characteristics before the first chemotherapy cycle (Table 2). Thus, when treating patients with breast cancer, oncologists should consider the effect of chemotherapy on frailty on all patients, both those younger and older than 65 years of age.

This study had several strengths. First, even though this was a younger cohort (mean age 53 years), we were able to replicate the findings of multiple studies on the association between cellular markers of inflammation and frailty. In addition, this study used available data from a cohort study that enrolled participants from multiple community oncology sites within the USA, making our findings generalizable to patients seen in oncology clinics within community settings that traditionally treat the majority of patients with cancer. Third, this was a large (*n* = 581) longitudinal study with measures obtained at multiple time points, allowing us to observe longitudinal changes in the cellular markers of inflammation as well as changes in frailty.

Our study also has limitations. We utilized a modified Fried’s frailty score due to the inability to measure unintentional weight loss. However, even with the use of the modified frailty criteria, we were able to corroborate previous findings of an association between inflammation and frailty. Given the complexity of the components and functions of the immune system, future work evaluating the role of cellular markers of inflammation on chronic chemotherapy-induced frailty should use immunoprofiling techniques, such as multiplex immunofluorescence, genomics, and/or proteomics.

## Conclusions

In patients with breast cancer receiving chemotherapy, cellular markers of inflammation are associated with acute but not persistent frailty. Immune cell counts may help clinicians identify patients at risk of frailty during chemotherapy. Additional research is needed to understand how changes in these immune cell profiles contribute to frailty and to determine the individual contributions of specific immune subsets.

## Supplementary Information

**Additional file 1:** **Table S1.** Association of Pre-Chemotherapy Cell Counts with Pre-Chemotherapy Frailty also controlling for the number of days between pre-chemotherapy lab draw and start of chemotherapy. **Table S2.** Association of Change in Cell Counts (Pre to Post-Chemotherapy) with Post-Chemotherapy Frailty also controlling for the number of days between post-chemotherapy lab draw and end of chemotherapy. **Table S3.** Association of Change in Cell Counts (Pre to Post-Chemotherapy) with Post-Chemotherapy Frailty in patients who received growth factor with chemotherapy. **Table S4.** Association of Pre-Chemotherapy Cell Counts with Frailty at Post-Chemotherapy and 6 Month Post-Chemotherapy Time-Points in Patients with Breast Cancer. **Table S5.** Association of Pre-Chemotherapy Cell Counts with Frailty at Post-Chemotherapy and 6 Month Post-Chemotherapy Time-Points in Patients with Breast Cancer in patients who received growth factor with chemotherapy. **Table S6.** Association of Change in Cell Counts (Pre to Post-Chemotherapy) with 6-Month Post-Chemotherapy Frailty.

## Data Availability

Data may be available upon request.
