# Supplementary material for: The longitudinal relationship between immune cell profiles and frailty in patients with breast cancer receiving chemotherapy
Source: Breast Cancer Res. 2021 Feb 5;23:19. doi: 10.1186/s13058-021-01388-w (PMC7863416; doi:10.1186/s13058-021-01388-w)
Supplement: Supplementary file 1 — Additional file 1: Table S1. Association of Pre-Chemotherapy Cell Counts with Pre-Chemotherapy Frailty also controlling for the number of days between pre-chemotherapy lab draw and start of chemotherapy. Table S2. Association of Change in Cell Counts (Pre to Post-Chemotherapy) with Post-Chemotherapy Frailty also controlling for the number of days between post-chemotherapy lab draw and end of chemotherapy. Table S3. Association of Change in Cell Counts (Pre to Post-Chemotherapy) with Post-Chemotherapy Frailty in patients who received growth factor with chemotherapy. Table S4. Association of Pre-Chemotherapy Cell Counts with Frailty at Post-Chemotherapy and 6 Month Post-Chemotherapy Time-Points in Patients with Breast Cancer. Table S5. Association of Pre-Chemotherapy Cell Counts with Frailty at Post-Chemotherapy and 6 Month Post-Chemotherapy Time-Points in Patients with Breast Cancer in patients who received growth factor with chemotherapy. Table S6. Association of Change in Cell Counts (Pre to Post-Chemotherapy) with 6-Month Post-Chemotherapy Frailty. [file 13058_2021_1388_MOESM1_ESM.docx]

| ***Supplementary Table 1:*** Association of Pre-Chemotherapy Cell Counts and Pre-Chemotherapy Frailty ***also controlling for the number of days between pre-chemotherapy lab draw and start of chemotherapy*** *β (SE)* | | | | | | |
| --- | --- | --- | --- | --- | --- | --- |
| **Pre-Chemotherapy** | **Pre-Chemotherapy Frailty Score** | | | | |  |
| Neutrophils | **0.039**** |  |  |  |  |  |
|  | **(0.019)** |  |  |  |  |  |
| Lymphocytes |  | 0.044 |  |  |  |  |
|  |  | (0.055) |  |  |  |  |
| Monocytes |  |  | 0.040 |  |  |  |
|  |  |  | (0.092) |  |  |  |
| NLR |  |  |  | **0.041**** |  |  |
|  |  |  |  | **(0.017)** |  |  |
| LMR |  |  |  |  | 0.001 |  |
|  |  |  |  |  | (0.002) |  |
| WBC |  |  |  |  |  | **0.039**** |
|  |  |  |  |  |  | **(0.017)** |
| Age: 50-64 | -0.147 | -0.140 | -0.130 | -0.142 | -0.140 | -0.118 |
|  | (0.096) | (0.097) | (0.096) | (0.097) | (0.096) | (0.091) |
| 65+ | 0.024 | 0.024 | 0.010 | 0.027 | 0.027 | 0.007 |
|  | (0.072) | (0.072) | (0.072) | (0.072) | (0.072) | (0.068) |
| White (Yes=1) | -0.176** | -0.174** | -0.152 | -0.179** | -0.159 | -0.154 |
|  | (0.087) | (0.087) | (0.086) | (0.087) | (0.088) | (0.081) |
| Married (Yes=1) | -0.124** | -0.117 | -0.122** | -0.129** | -0.130** | -0.116** |
|  | (0.059) | (0.060) | (0.059) | (0.059) | (0.059) | (0.056) |
| Some College or Above=1 | -0.151** | -0.126** | -0.130** | -0.146** | -0.125** | -0.148*** |
|  | (0.061) | (0.060) | (0.060) | (0.060) | (0.060) | (0.057) |
| # Days between Pre-chemo labs and start of chemotherapy | 0.0002 | -9.4^e-5^ | -7.5^e-5^ | 3.3^e-5^ | 9.3^e-5^ | -0.002 |
|  | (0.003) | (0.003) | (0.003) | (0.003) | (0.003) | 0.003 |
| Constant | 1.330*** | 1.420*** | 1.468*** | 1.401*** | 1.482*** | 1.216*** |
|  | (0.138) | (0.152) | (0.114) | (0.115) | (0.105) | (0.156) |
| Observations | 472 | 475 | 476 | 471 | 467 | 515 |
| R-squared | 0.045 | 0.035 | 0.033 | 0.047 | 0.034 | 0.044 |
| Notes: Linear regression models were used to evaluate the association between cell counts and frailty, controlling for age (below 50, 50-64 versus ≥65 years), race (white vs non-white), marital status (married versus other), education (≥ some college versus ≤ high school).  *significant at 10%; **significant at 5%; ***significant at 1%.  NLR: Neutrophil to Lymphocyte ratio; LMR: Lymphocyte to Monocyte ratio | | | | | | |

| ***Supplementary Table 2:*** Association of Change in Cell Counts (Pre to Post-Chemotherapy) and with Post-Chemotherapy Frailty ***also controlling for the number of days between post-chemotherapy lab draw and end of chemotherapy*** | | | | | | |  |
| --- | --- | --- | --- | --- | --- | --- | --- |
| **Change in Cell Counts** | **Post-Chemotherapy Frailty Score:** *β (SE)* | | | | | |  |
| Neutrophils | **0.024**** |  |  |  |  |  | |
|  | **(0.011)** |  |  |  |  |  | |
| Lymphocytes |  | 0.046 |  |  |  |  | |
|  |  | (0.032) |  |  |  |  | |
| Monocytes |  |  | 0.024 |  |  |  | |
|  |  |  | (0.074) |  |  |  | |
| NLR |  |  |  | **0.029***** |  |  | |
|  |  |  |  | **(0.009)** |  |  | |
| LMR |  |  |  |  | -0.003* |  | |
|  |  |  |  |  | (0.002) |  | |
| WBC |  |  |  |  |  | **0.021**** | |
|  |  |  |  |  |  | **(0.010)** | |
| Baseline Frailty | 0.326*** | 0.321*** | 0.319*** | 0.335*** | 0.325*** | 0.311*** | |
|  | (0.047) | (0.047) | (0.047) | (0.048) | (0.049) | (0.046) | |
| Age: 50-64 | 0.141 | 0.145 | 0.138 | 0.109 | 0.150 | 0.099 | |
|  | (0.098) | (0.099) | (0.098) | (0.101) | (0.102) | (0.095) | |
| 65+ | 0.042 | 0.044 | 0.042 | 0.049 | 0.029 | 0.063 | |
|  | (0.073) | (0.073) | (0.073) | (0.074) | (0.076) | (0.070) | |
| White (Yes=1) | 0.042 | 0.061 | 0.032 | 0.049 | 0.073 | 0.018 | |
|  | (0.089) | (0.089) | (0.088) | (0.089) | (0.091) | (0.084) | |
| Married (Yes=1) | -0.159*** | -0.155** | -0.157*** | -0.142** | -0.136** | -0.146** | |
|  | (0.060) | (0.060) | (0.060) | (0.061) | (0.062) | (0.059) | |
| Some College or Above=1 | 0.070 | 0.063 | 0.052 | 0.098 | 0.068 | 0.085 | |
|  | (0.061) | (0.061) | (0.061) | (0.063) | (0.064) | (0.059) | |
| # Days between date of post-chemo lab draw and end of chemotherapy | -0.004** | -0.005** | -0.005** | -0.003 | -0.004 | -0.004** | |
|  | 0.002 | 0.002 | 0.002 | 0.002 | 0.002 | 0.002 | |
| Constant | 1.647*** | 1.715*** | 1.718*** | 1.531*** | 1.621** | 1.732*** | |
|  | (0.126) | (0.129) | (0.125) | (0.131) | (0.130) | (0.119) | |
| Observations | 452 | 451 | 454 | 436 | 424 | 502 | |
| R-squared | 0.141 | 0.133 | 0.126 | 0.151 | 0.132 | 0.124 | |
| Notes: Linear regression models were used to evaluate the association between cell counts and frailty, controlling for age (below 50, 50-64 versus ≥65 years), race (white vs non-white), marital status (married versus other), education (≥ some college versus ≤ high school).  *significant at 10%; **significant at 5%; ***significant at 1%.  NLR: Neutrophil to Lymphocyte ratio; LMR: Lymphocyte to Monocyte ratio | | | | | | |  |

| ***Supplementary Table 3:*** Association of Change in Cell Counts (Pre to Post-Chemotherapy) and with Post-Chemotherapy Frailty ***in patients who received growth factor with chemotherapy*** | | | | | | |
| --- | --- | --- | --- | --- | --- | --- |
| **Change in Cell Counts** | **Post-Chemotherapy Frailty Score:** *β (SE)* | | | | | |
| Neutrophils | **0.023**** |  |  |  |  |  |
|  | **(0.011)** |  |  |  |  |  |
| Lymphocytes |  | 0.049 |  |  |  |  |
|  |  | (0.032) |  |  |  |  |
| Monocytes |  |  | 0.029 |  |  |  |
|  |  |  | (0.074) |  |  |  |
| NLR |  |  |  | **0.031***** |  |  |
|  |  |  |  | **(0.010)** |  |  |
| LMR |  |  |  |  | -0.003* |  |
|  |  |  |  |  | (0.002) |  |
| WBC |  |  |  |  |  | **0.023**** |
|  |  |  |  |  |  | **(0.010)** |
| Baseline Frailty | 0.316*** | 0.315*** | 0.311*** | 0.328*** | 0.322*** | 0.290*** |
|  | (0.049) | (0.049) | (0.049) | (0.050) | (0.051) | (0.049) |
| Age: 50-64 | 0.211* | 0.205* | 0.191 | 0.213* | 0.195 | 0.192 |
|  | (0.123) | (0.124) | (0.124) | (0.125) | (0.129) | (0.120) |
| 65+ | 0.294* | 0.295* | 0.267 | 0.275 | 0.339* | 0.194 |
|  | (0.171) | (0.173) | (0.173) | (0.176) | (0.178) | (0.168) |
| White (Yes=1) | 0.220 | 0.267 | 0.216 | 0.156 | 0.227 | 0.134 |
|  | (0.194) | (0.194) | (0.192) | (0.196) | (0.200) | (0.190) |
| Married (Yes=1) | -0.303** | -0.288** | -0.287** | -0.251* | -0.236* | -0.276** |
|  | (0.126) | (0.128) | (0.128) | (0.129) | (0.133) | (0.125) |
| Some College or Above=1 | 0.111 | 0.110 | 0.092 | 0.170 | 0.130 | 0.167 |
|  | (0.129) | (0.129) | (0.130) | (0.132) | (0.133) | (0.126) |
| Constant | 1.356*** | 1.367*** | 1.402*** | 1.244*** | 1.269*** | 1.490*** |
|  | (0.249) | (0.251) | (0.250) | (0.255) | (0.258) | (0.248) |
| Observations | 394 | 393 | 395 | 375 | 365 | 435 |
| R-squared | 0.137 | 0.129 | 0.118 | 0.152 | 0.131 | 0.111 |
| Notes: Linear regression models were used to evaluate the association between cell counts and frailty, controlling for age (below 50, 50-64 versus ≥65 years), race (white vs non-white), marital status (married versus other), education (≥ some college versus ≤ high school).  *significant at 10%; **significant at 5%; ***significant at 1%.  NLR: Neutrophil to Lymphocyte ratio; LMR: Lymphocyte to Monocyte ratio | | | | | | |

| ***Supplementary Table 4:*** Association of Pre-Chemotherapy Cell Counts with Frailty at Post-Chemotherapy and 6 Month Post-Chemotherapy Time-Points in Patients with Breast Cancer | | | | | | | | | | | |  |
| --- | --- | --- | --- | --- | --- | --- | --- | --- | --- | --- | --- | --- |
| **Pre-Chemotherapy** | **Post-Chemotherapy Frailty Score:** *β (SE)* | | | | | | **6 Month Post-Chemotherapy Frailty Score:** *β (SE)* | | | | | |
| Neutrophils | -0.026 |  |  |  |  |  | -0.019 |  |  |  |  |  |
|  | (0.019) |  |  |  |  |  | (0.017) |  |  |  |  |  |
| Lymphocytes |  | -0.022 |  |  |  |  |  | 0.066 |  |  |  |  |
|  |  | (0.053) |  |  |  |  |  | (0.048) |  |  |  |  |
| Monocytes |  |  | -0.030 |  |  |  |  |  | -0.013 |  |  |  |
|  |  |  | (0.092) |  |  |  |  |  | (0.082) |  |  |  |
| NLR |  |  |  | -0.019 |  |  |  |  |  | -0.032* |  |  |
|  |  |  |  | (0.018) |  |  |  |  |  | (0.017) |  |  |
| LMR |  |  |  |  | 0.003* |  |  |  |  |  | -0.001 |  |
|  |  |  |  |  | (0.002) |  |  |  |  |  | (0.001) |  |
| WBC |  |  |  |  |  | -0.018 |  |  |  |  |  | -0.005 |
|  |  |  |  |  |  | (0.016) |  |  |  |  |  | (0.015) |
| Baseline Frailty | 0.327*** | 0.324*** | 0.312*** | 0.333*** | 0.318*** | 0.299*** | 0.452*** | 0.445*** | 0.448*** | 0.460*** | 0.453*** | 0.444*** |
|  | (0.047) | (0.046) | (0.046) | (0.047) | (0.048) | (0.046) | (0.044) | (0.043) | (0.043) | (0.045) | (0.045) | (0.042) |
| Age: 50-64 | 0.203* | 0.212* | 0.215* | 0.180 | 0.178 | 0.226** | 0.204* | 0.212** | 0.211** | 0.201* | 0.189* | 0.219** |
|  | (0.115) | (0.114) | (0.115) | (0.117) | (0.117) | (0.111) | (0.105) | (0.105) | (0.105) | (0.107) | (0.108) | (0.101) |
| 65+ | 0.288* | 0.282* | 0.263 | 0.254 | 0.247 | 0.257 | 0.088 | 0.106 | 0.095 | 0.069 | 0.089 | 0.083 |
|  | (0.161) | (0.161) | (0.160) | (0.165) | (0.164) | (0.156) | (0.149) | (0.149) | (0.148) | (0.153) | (0.152) | (0.143) |
| White | 0.156 | 0.153 | 0.095 | 0.171 | 0.181 | 0.069 | -0.059 | -0.073 | -0.076 | -0.026 | -0.024 | -0.142 |
|  | (0.176) | (0.176) | (0.174) | (0.181) | (0.182) | (0.170) | (0.171) | (0.170) | (0.167) | (0.175) | (0.178) | (0.161) |
| Married | -0.341*** | -0.345*** | -0.353*** | -0.329*** | -0.339*** | -0.320*** | -0.247** | -0.239** | -0.264** | -0.240** | -0.263** | -0.275** |
|  | (0.119) | (0.120) | (0.119) | (0.122) | (0.122) | (0.117) | (0.110) | (0.111) | (0.110) | (0.113) | (0.113) | (0.107) |
| Some College or Above | 0.161 | 0.140 | 0.128 | 0.183 | 0.154 | 0.186 | -0.087 | -0.080 | -0.115 | -0.063 | -0.080 | -0.091 |
|  | (0.123) | (0.122) | (0.122) | (0.125) | (0.125) | (0.118) | (0.113) | (0.111) | (0.111) | (0.114) | (0.115) | (0.107) |
| Constant | 1.521*** | 1.467*** | 1.524*** | 1.410*** | 1.386*** | 1.645*** | 1.017*** | 0.808*** | 0.990*** | 0.962*** | 0.903*** | 1.073*** |
|  | (0.241) | (0.257) | (0.233) | (0.240) | (0.237) | (0.251) | (0.229) | (0.242) | (0.220) | (0.229) | (0.228) | (0.234) |
| Observations | 474 | 477 | 478 | 461 | 457 | 511 | 445 | 448 | 449 | 432 | 428 | 480 |
| R-squared | 0.125 | 0.123 | 0.117 | 0.125 | 0.125 | 0.106 | 0.225 | 0.226 | 0.225 | 0.230 | 0.224 | 0.223 |
| Notes: Linear regression models were used to evaluate the association between cell counts and frailty, controlling for baseline frailty, age (below 50, 50-64 versus ≥65 years), race (white vs non-white), marital status (married versus other), and education (≥ some college versus ≤ high school)  Beta Coefficient (β); Standard Deviation (SD); Neutrophil to Lymphocyte Ratio (NLR); Lymphocyte to Monocyte Ratio (LMR); White Blood Cell (WBC)  *significant at 10%; **significant at 5%; ***significant at 1%. | | | | | | | | | | | | |

| ***Supplementary Table 5:*** Association of Pre-Chemotherapy Cell Counts with Frailty at Post-Chemotherapy and 6 Month Post-Chemotherapy Time-Points in Patients with Breast Cancer ***in patients who received growth factor with chemotherapy*** | | | | | | | | | | | | |
| --- | --- | --- | --- | --- | --- | --- | --- | --- | --- | --- | --- | --- |
| **Pre-Chemotherapy** | **Post-Chemotherapy Frailty Score:** *β (SE)* | | | | | | **6 Month Post-Chemotherapy Frailty Score:** *β (SE)* | | | | | |
| Neutrophils | -0.018 |  |  |  |  |  | -0.024 |  |  |  |  |  |
|  | (0.021) |  |  |  |  |  | (0.019) |  |  |  |  |  |
| Lymphocytes |  | -0.044 |  |  |  |  |  | 0.044 |  |  |  |  |
|  |  | (0.056) |  |  |  |  |  | (0.051) |  |  |  |  |
| Monocytes |  |  | -0.037 |  |  |  |  |  | -0.013 |  |  |  |
|  |  |  | (0.092) |  |  |  |  |  | (0.083) |  |  |  |
| NLR |  |  |  | -0.012 |  |  |  |  |  | -0.032* |  |  |
|  |  |  |  | (0.019) |  |  |  |  |  | (0.018) |  |  |
| LMR |  |  |  |  | 0.003* |  |  |  |  |  | -0.001 |  |
|  |  |  |  |  | (0.002) |  |  |  |  |  | (0.001) |  |
| WBC |  |  |  |  |  | -0.017 |  |  |  |  |  | -0.011 |
|  |  |  |  |  |  | (0.018) |  |  |  |  |  | (0.016) |
| Baseline Frailty | 0.323*** | 0.327*** | 0.313*** | 0.331*** | 0.316*** | 0.294*** | 0.438*** | 0.433*** | 0.438*** | 0.444*** | 0.438*** | 0.435*** |
|  | (0.050) | (0.049) | (0.049) | (0.051) | (0.051) | (0.049) | (0.047) | (0.047) | (0.047) | (0.048) | (0.048) | (0.045) |
| Age: 50-64 | 0.186 | 0.184 | 0.188 | 0.159 | 0.151 | 0.208* | 0.227** | 0.233** | 0.221* | 0.229* | 0.212* | 0.248** |
|  | (0.124) | (0.123) | (0.124) | (0.126) | (0.126) | (0.120) | (0.115) | (0.115) | (0.115) | (0.117) | (0.118) | (0.110) |
| 65+ | 0.249 | 0.234 | 0.216 | 0.238 | 0.230 | 0.206 | 0.058 | 0.065 | 0.062 | 0.051 | 0.069 | 0.063 |
|  | (0.173) | (0.173) | (0.172) | (0.177) | (0.175) | (0.169) | (0.162) | (0.162) | (0.161) | (0.166) | (0.165) | (0.155) |
| White | 0.274 | 0.271 | 0.225 | 0.254 | 0.270 | 0.173 | 0.046 | 0.040 | 0.064 | 0.070 | 0.081 | -0.031 |
|  | (0.197) | (0.197) | (0.194) | (0.201) | (0.203) | (0.190) | (0.194) | (0.193) | (0.190) | (0.198) | (0.201) | (0.182) |
| Married | -0.306** | -0.308** | -0.306** | -0.299** | -0.311** | -0.281** | -0.217* | -0.210* | -0.220* | -0.214* | -0.235* | -0.235** |
|  | (0.128) | (0.128) | (0.128) | (0.131) | (0.131) | (0.126) | (0.119) | (0.120) | (0.119) | (0.123) | (0.123) | (0.115) |
| Some College or Above | 0.117 | 0.101 | 0.098 | 0.151 | 0.136 | 0.164 | -0.110 | -0.117 | -0.121 | -0.084 | -0.100 | -0.114 |
|  | (0.130) | (0.130) | (0.130) | (0.133) | (0.132) | (0.127) | (0.121) | (0.120) | (0.120) | (0.123) | (0.123) | (0.116) |
| Constant | 1.410*** | 1.431*** | 1.427*** | 1.345*** | 1.329*** | 1.558*** | 0.935*** | 0.754*** | 0.829*** | 0.864*** | 0.799*** | 0.981*** |
|  | (0.266) | (0.282) | (0.257) | (0.263) | (0.259) | (0.281) | (0.256) | (0.269) | (0.247) | (0.255) | (0.253) | (0.263) |
| Observations | 406 | 408 | 409 | 394 | 392 | 437 | 379 | 381 | 382 | 367 | 365 | 409 |
| R-squared | 0.123 | 0.125 | 0.116 | 0.125 | 0.126 | 0.102 | 0.223 | 0.222 | 0.222 | 0.226 | 0.220 | 0.222 |
| Notes: Linear regression models were used to evaluate the association between cell counts and frailty, controlling for baseline frailty, age (below 50, 50-64 versus ≥65 years), race (white vs non-white), marital status (married versus other), and education (≥ some college versus ≤ high school)  Beta Coefficient (β); Standard Deviation (SD); Neutrophil to Lymphocyte Ratio (NLR); Lymphocyte to Monocyte Ratio (LMR); White Blood Cell (WBC)  *significant at 10%; **significant at 5%; ***significant at 1%. | | | | | | | | | | | | |

| ***Supplementary Table 6:*** Association of Change in Cell Counts (Pre to Post-Chemotherapy) and with 6-Month Post-Chemotherapy Frailty  *β (SE)* | | | | | | |
| --- | --- | --- | --- | --- | --- | --- |
| **Change in cell count Pre- and Post-Chemotherapy** | **6-month Post-Chemotherapy frailty score** | | | | | |
| Neutrophils | 0.008 |  |  |  |  |  |
|  | (0.010) |  |  |  |  |  |
| Lymphocytes |  | -0.012 |  |  |  |  |
|  |  | (0.029) |  |  |  |  |
| Monocytes |  |  | 0.061 |  |  |  |
|  |  |  | (0.067) |  |  |  |
| NLR |  |  |  | 0.004 |  |  |
|  |  |  |  | (0.009) |  |  |
| LMR |  |  |  |  | 0.001 |  |
|  |  |  |  |  | (0.001) |  |
| WBC |  |  |  |  |  | 0.004 |
|  |  |  |  |  |  | (0.008) |
| Baseline Frailty | 0.456*** | 0.459*** | 0.455*** | 0.455*** | 0.461*** | 0.445*** |
|  | (0.045) | (0.045) | (0.044) | (0.046) | (0.047) | (0.042) |
| Age: 50-64 | 0.237** | 0.243** | 0.255** | 0.248** | 0.214* | 0.235** |
|  | (0.108) | (0.107) | (0.107) | (0.111) | (0.112) | (0.101) |
| 65+ | 0.115 | 0.145 | 0.141 | 0.132 | 0.171 | 0.095 |
|  | (0.151) | (0.151) | (0.151) | (0.158) | (0.158) | (0.143) |
| White (Yes=1) | -0.040 | -0.034 | -0.044 | -0.013 | 0.002 | -0.151 |
|  | (0.174) | (0.173) | (0.169) | (0.180) | (0.182) | (0.161) |
| Married (Yes=1) | -0.258** | -0.260** | -0.276** | -0.271** | -0.270** | -0.273** |
|  | (0.112) | (0.113) | (0.112) | (0.117) | (0.118) | (0.107) |
| Some College or Above=1 | -0.070 | -0.068 | -0.090 | -0.057 | -0.058 | -0.077 |
|  | (0.116) | (0.115) | (0.114) | (0.120) | (0.120) | (0.109) |
| Constant | 0.880*** | 0.855*** | 0.910*** | 0.840*** | 0.824*** | 1.018*** |
|  | (0.225) | (0.226) | (0.220) | (0.236) | (0.236) | (0.211) |
| Observations | 429 | 429 | 431 | 409 | 397 | 475 |
| R-squared | 0.229 | 0.229 | 0.230 | 0.224 | 0.227 | 0.225 |
| Notes: Linear regression models were used to evaluate the association between cell counts and frailty, controlling for baseline frailty, age (below 50, 50-64 versus ≥65 years), race (white vs non-white), marital status (married versus other), and education (≥ some college versus ≤ high school)  Beta Coefficient (β); Standard Deviation (SD); Neutrophil to Lymphocyte Ratio (NLR); Lymphocyte to Monocyte Ratio (LMR); White Blood Cell (WBC)  *significant at 10%; **significant at 5%; ***significant at 1%. | | | | | | |
